# Feasibility and Acceptability of a Mobile Technology Intervention to Support Postabortion Care in British Columbia: Phase I

**DOI:** 10.2196/13387

**Published:** 2019-05-29

**Authors:** Roopan Gill, Gina Ogilvie, Wendy V Norman, Brian Fitzsimmons, Ciana Maher, Regina Renner

**Affiliations:** 1 Department of Obstetrics and Gynaecology Women's Health Research Institute University of British Columbia Vancouver, BC Canada

**Keywords:** mHealth, family planning, abortion, induced, sexual health, digital health, user-centered design

## Abstract

**Background:**

Over 30% of women in Canada undergo an abortion. Despite the prevalence of the procedure, stigma surrounding abortion in Canada leads to barriers for women to access this service. The vast majority of care is concentrated in urban settings. There is evidence to support utilization of innovative mobile and other technology solutions to empower women to safely and effectively self-manage aspects of the abortion process. This study is part 1 of a 3-phase study that utilizes user-centered design methodology to develop a digital health solution to specifically support follow-up after an induced surgical abortion.

**Objective:**

This study aimed to (1) understand how women at 3 surgical abortion clinics in an urban center of British Columbia utilize their mobile phones to access health care information and (2) understand women’s preferences of content and design of an intervention that will support follow-up care after an induced abortion, including contraceptive use.

**Methods:**

The study design was based on *development-evaluation-implementation* process from Medical Research Council Framework for Complex Medical Interventions. This was a mixed-methods formative study. Women (aged 14-45 years) were recruited from 3 urban abortion facilities in British Columbia who underwent an induced abortion. Adaptation of validated surveys and using the technology acceptance model and theory of reasoned action, a cross-sectional survey was designed. Interview topics included demographic information; type of wireless device used; cell phone usage; acceptable information to include in a mobile intervention to support women’s abortion care; willingness to use a mobile phone to obtain reproductive health information; optimal strategies to use a mobile intervention to support women; understand preferences for health information resources; and design qualities in a mobile intervention important for ease of use, privacy, and security. Responses to questions in the survey were summarized using descriptive statistics. Qualitative analysis was conducted with NVivo using a thematic analysis approach. This study was approved by the local ethics board.

**Results:**

A waiting-room survey was completed by 50 participants, and semistructured interviews were completed with 8 participants. The average age of participants was 26 years. Furthermore, 94% (47/50) owned a smartphone, 85% (41/48) used their personal phones to go online, and 85% would use their cell phone to assist in clinical care. Qualitative analysis demonstrated that women prefer a comprehensive website that included secure email or text notifications to provide tools and resources for emotional well-being, contraceptive decision making, general sexual health, and postprocedure care.

**Conclusions:**

A community-based mixed-methods approach allowed us to understand how women use their cell phones and what women desire in a mobile intervention to support their postabortion care. The findings from this formative phase will assist in the development and testing of a mobile intervention to support follow-up care after an induced surgical abortion.

## Introduction

### Background

Despite abortion being without any criminal restriction in Canada and over 30% of women in Canada undergo an abortion, recent studies suggest that women who have an abortion experience isolation, particularly for those who travel far distances, perceived need for secrecy, feelings of stigma, and other social factors, suggesting that access to the procedure is only one aspect of what it means to deliver quality abortion care [1,2]. Specifically, studies have demonstrated that the dynamics of the political and social climate in Canada can lead to both externalized and internalized stigma for individuals who undergo an abortion [1,3,4]. Despite global guidance that states that follow-up is not necessary after an induced surgical abortion, there is evidence documenting women’s desire for postabortion support [5-7]. Opportunities to use innovations using digital solutions for abortion care can support women to self-manage part, if not all, of the abortion process [6,8-11]. More importantly, evidence has pointed to its potential to provide follow-up support, particularly for women who live in rural and remote areas [4].

The intersection of digital health with self-care is moving rapidly and being utilized in various aspects of health care [12-17]. The New England Journal of Medicine published a special report on Telehealth—an example of digital health—in the United States, highlighting its utility and future. In 2016, Kaiser Permanente of Northern California reported that its virtual (email, telephone, and video) communications had exceeded in-person visits [18]. Similarly, research supports the safe and effective use of digital health solutions for provision of medical abortion care globally [19-25]. Evidence supports its safe, effective, and acceptable utility in provision of abortion. Studies utilizing hotlines, text messaging, and mobile apps in Cambodia, Indonesia, and South Africa are just a few examples of how digital health is being utilized to improve the abortion experience for women who face barriers to abortion care globally [14,20,26,27]. Despite the development and implementation of mobile health (mHealth) innovations for family planning, research is limited in understanding the follow-up needs of women who undergo an abortion, how they would perceive a digital health tool to support them, and more importantly, engage them as active participants in the design process.

### Objectives

Given the existing evidence in support of mHealth for family planning innovations, we aimed to determine if a mobile technology intervention would be acceptable and feasible to women to support follow-up care after first and second trimester surgical abortion. This study was designed and conducted before the availability of mifepristone for medical abortion in Canada, and thus, women undergoing a surgical abortion were the focus population for this study [28]. We developed a 3-phased study based on user-centered design and the *development- evaluation-implementation* process from Medical Research Council Frameworks for Complex Interventions [29]. Phase I was a mixed-methods formative design to understand how women at 3 surgical abortion clinics utilize their mobile phones to access health care information and to understand their preferences of content and design of a mobile intervention that will support follow-up care after a surgical abortion. Phase II was the design, development, and usability testing aspect of the study, and finally, phase III was a prospective mixed-methods pilot study to determine the feasibility and acceptability of the mobile intervention to support follow-up care after a surgical abortion. This study is the first to utilize mHealth and user-centered design in Canada as a novel approach to support follow-up care for women who undergo surgical abortion. The focus of this manuscript is phase I methodology and results.

## Methods

### Participants

Participants were recruited from publicly funded abortion clinics, 3 in urban settings and 1 in a rural site within British Columbia, Canada. The eligibility criteria were that the participant should (1) have consented to undergo a first or second trimester surgical abortion procedure, (2) be able to read and write English, (3) be able to participate in study procedures, and (4) be aged ≥14 years. The participants were excluded if they were (1) attending the clinics because of fetal anomaly or miscarriage, (2) undergoing medical abortion, (3) in a situation that may be dangerous to utilize a mobile intervention, and (4) unable to provide consent to participate. To elicit whether a woman was in a dangerous situation, counselors asked patients as part of routine care if they felt safe in their current relationships. In cases where risk is identified, counselors provided resources and would refer to the appropriate provider or service.

### Theoretical Framework

The study design and instruments were developed using the technology acceptance model (TAM) and theory of reasoned action (TRA). These theories have been validated in studies for the development of mHealth solutions in low- and middle-income countries [27,30,31]. TAM identifies 2 distinct attitudes that could predict the adoption of a new information system: perceived ease of use and usefulness of the system. TRA states that a person’s behavior can be determined by 3 factors: (1) subjective norms, defined as whether or not people important to the individual believe they should perform the behavior or not; (2) individual’s attitude toward the behavior; and (3) an individual’s intention to engage in a behavior [30-32]. The study instruments for all 3 phases were developed using these theories based on validated survey tools [13,31,33,34]. Specifically, these tools have been utilized for formative research on developing digital health solutions specific to health behaviors.

### Study Design

The overall study design is a mixed-methods user-centered design approach with 3 phases based on the *development-evaluation-implementation* process from the Medical Research Council Frameworks for Complex Medical Interventions [35]. Phase I is a community-based mixed-methods study conducted in 3 urban clinics in Vancouver, British Columbia, between May and August 2017 with the goal of understanding how women utilize technology to access health care information and their preferences of content and design of a mobile intervention to support follow-up care after an induced surgical abortion. The quantitative component of the study included an anonymous survey that was adapted from validated surveys from studies conducted for mHealth interventions and contraception use [13,31,33,36]. The survey asked questions about (1) how they use technology and their preferences for its use in clinical care and follow-up after an abortion, (2) mobile device use, privacy, and security, and (3) their past experience with contraception. The qualitative interview guide included topics on the types of wireless device used, technology engagement, acceptable information to include in a mobile intervention to support women’s abortion care, willingness to use a mobile phone to obtain reproductive health information, optimal strategies to use the mobile intervention to support women, understanding preferences for health information resources, design qualities of mobile intervention, and privacy and security. Counselors at each of the abortion clinics obtained consent from eligible participants and distributed the survey for participants to complete in the waiting room or counselors’ office before going for their procedure. Embedded within the survey, we asked participants whether they consented to being contacted within a week of their visit by our research coordinator to participate in an optional semistructured individual interview for the qualitative component of this study. Participants who consented to a follow-up telephone call received information regarding the qualitative component of the study, and if they were interested in participating, a convenient time was set for the principal investigator or the research coordinator to interview participants over the phone. Verbal consent was obtained at the start of the audio-recorded interviews, which lasted approximately 45 min each. Participants received a Can $25 gift card for participating in the semistructured interview. A convenience sample of approximately 50 participants for the quantitative component of the study was desired and, for the qualitative component, 10 to 20 participants or until saturation of themes was reached. Studies have suggested that thematic saturation could be reached with as few as 12 to 15 participants [37].

This study was approved by the Children’s and Women’s Research Ethics Board (H16-02823).

### Data Analysis

#### Phase I

Descriptive analysis of each variable from the waiting room survey was reported as a mean (SD) or median for continuous variables and count (%) for categorical variables. The semistructured interview transcripts were uploaded to NVivo 11 (QSR International Pty Ltd) and read by 2 researchers. Inductive analysis was done to identify emerging themes, which were further refined through collaborative analysis with the first author and coinvestigator. Highlighted text was coded into nodes representing similar or repeating ideas. Some text was coded to more than 1 node to reflect the number of ideas presented. Nodes were then grouped together in a respective theme and a thematic map was developed and discussed with the research team and triangulation of data with expert opinion was used to enhance the validity of the findings.

## Results

### Participant Characteristics

Table 1 provides the demographic profile of participants. A total of 50 participants were recruited, and of these participants, 78% were Canadian, 84% were under the age of 30 years, 69% had an annual income less than Can $35,000, and 36% had high school education, whereas 38% were between high school and a bachelor’s degree. There was variation in the distance traveled by participants, with 30% traveling less than 10 km, 22% traveling between 20 and 40 km, and 18% who traveled over 100 km. In addition, 94% (47/50) of participants reported owning a smartphone. Of the 50 participants who completed the survey, 25 consented to being contacted about the interview, and a total of 8 participants were recruited to participate in the semistructured individual phone interviews.

**Table 1:** Demographic profile of participants (N=50).

Demographic details		n (%)
**Birthplace**		
	Canada	39 (78)
	Other	11 (22)
**Age (years)**		
	18-25	27 (54)
	26-30	17 (34)
	31-37	6 (12)
**Annual income**		
	Can $0-15,000	16 (31)
	Can $15,000-35,000	18 (38)
	Can $35,000-55,000	8 (17)
	Can $55,000+	7 (13)
**Education**		
	High school	17 (36)
	Between high school and bachelor’s	19 (38)
	Bachelor’s or higher	13 (23)
**Distanced traveled to clinic**		
	<10 km	15 (30)
	10-20 km	5 (10)
	20-40 km	11 (22)
	40-60 km	6 (12)
	60-80 km	4 (8)
	100+ km	9 (18)
**Own cell phone**		
	Yes (basic)	3 (6)
	Yes (smartphone)	47 (94)

### Quantitative Results

**Figure 1:**
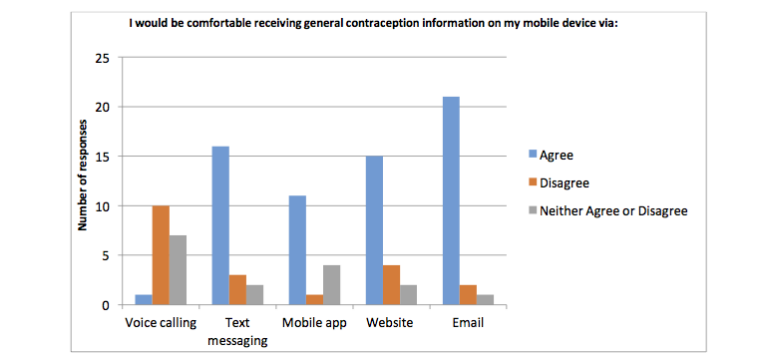
Number of participants who are comfortable receiving information about contraception via different modalities on their mobile device.

**Figure 2:**
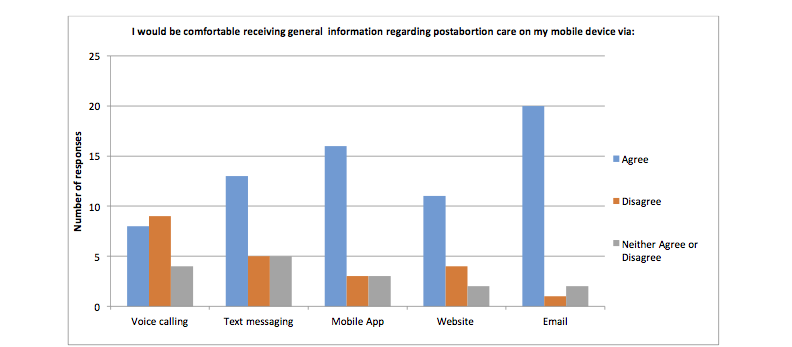
Number of participants who are comfortable receiving information about postabortion care via different modalities on their mobile device.

**Figure 3:**
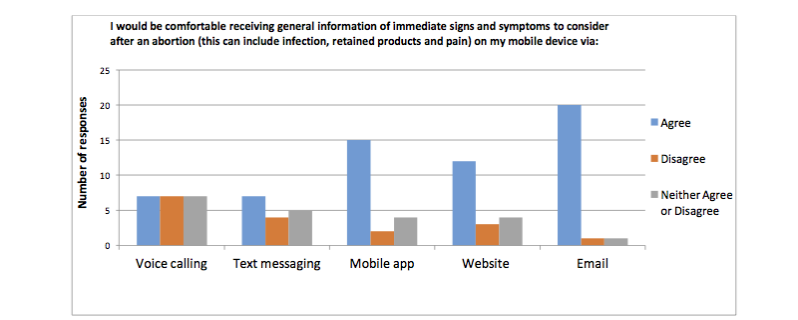
Number of participants who are comfortable receiving information about postoperative signs and symptoms via different modalities on their mobile device.

**Figure 4:**
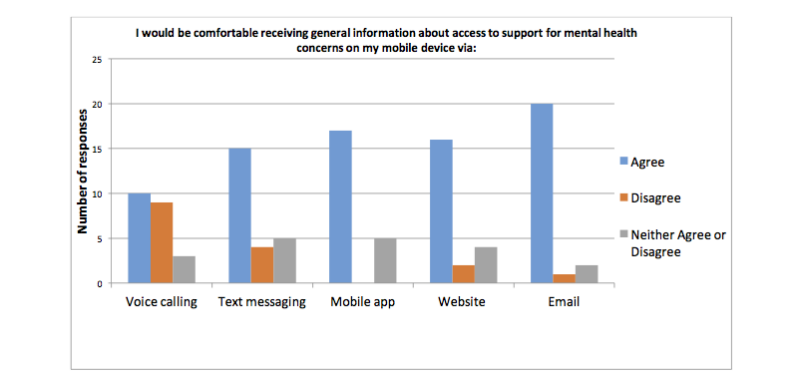
Number of participants who are comfortable receiving information about mental health via different modalities on their mobile device.

Results from the waiting room survey are summarized in Table 2. Specifically, 89% (42/47) of participants stated they use a smart phone most often out of a list of five commonly used devices, 92% (45/49) of participants’ phone plans included unlimited SMS plans, 92% (45/49) of participants use the internet on their phone. 88% (42/49) of the participants use their personal phones to go online, 85% (41/48) liked the idea of using a cell phone to assist in their clinical care and follow-up and 92% (45/49) of participants used mobile apps. Participants reported being more comfortable with receiving information about contraception, general postabortion care information, signs and symptoms after an abortion, mental health information and sexual health via email followed by website and mobile apps opposed to other modalities. Results are presented in Figures 1-4.

**Table 2:** Phase I survey results.

Survey questions (N=50)		n (%)
**Which device do you use?**		
	Basic mobile phone	4 (8)
	Smartphone	47 (94**)**
	Tablet	17 (34)
	Laptop	35 (70)
	Desktop computer	16 (32)
**Which device do you use the most? (N=47)**		
	Basic mobile phone	2 (4)
	Smartphone	42 (89)
	Tablet	0 (0)
	Laptop	2 (4)
	Desktop computer	0 (0)
	All of the above	1 (2)
**When you have question about your health, what do you use? (N=50)**		
	Internet	46 (92)
	Social media	3 (6)
	Mobile apps	3 (6)
	Friends	20 (40)
	Family	20 (40)
	Healthcare Provider	34 (68)
**What do you use the most often? (N=50)**		
	Internet	23 (46)
	Social media	5 (10)
	Mobile app	2 (4)
	Friends	2 (4)
	Family	2 (4)
	HP	6 (12)
**Does your cell phone plan include text messaging? (N=49)**		
	Yes, pay per text	0 (0)
	Yes, limited short message service (SMS)	2 (4)
	Yes, unlimited SMS	45 (91)
	No	1 (2)
	Don’t know	1 (2)
**Do you use the internet on your phone? (N=49)**		
	Yes	45 (91)
	No	3 (6)
	Don’t know	1 (2)
**Would like the idea of using cell phone to assist with follow-up care? (N=48)**		
	Yes	41 (85)
	No	3 (6)
	I don’t know	4 (8)
**How participants go online^a^ (N=49)**		
	Personal computer	34 (69)
	Work	7 (14)
	Family/friends’ computer	5 (10)
	Personal phone	42 (85)
	Internet café	1 (2)
	Family/friends’ phone	1 (2)
	All of the above	0 (0)
	I don’t go online	0 (0)
**Mobile app use (N=49)**		
	Yes	45 (92)
	No	3 (6)
	I don’t know	1 (2)
**Features that make a mobile app easy to use^a^ (N=49)**		
	Simple design	44 (90)
	Not overly time consuming	31 (63)
	Controlled push notifications	18 (37)
	Easy scrolling	28 (57)
	Link to other websites	4 (8)
	Log in by Facebook/Google+	16 (33)
	Integration with other apps	8/49 (16)
	I don’t know	2 (4)
**Frequency of messages from clinic (N=48)**		
	Daily	1 (2)
	Once/week	14 (29)
	Twice/week	8 (4)
	Once/month	25 (52)
**Prefer not to see the words abortion, birth control, and contraception in a text message (N=48)**		
	Yes, prefer if not used	35 (73)
	No, I don’t care if they are used	13 (27)

^a^Participants to *mark all that applied*.

**Figure 5:**
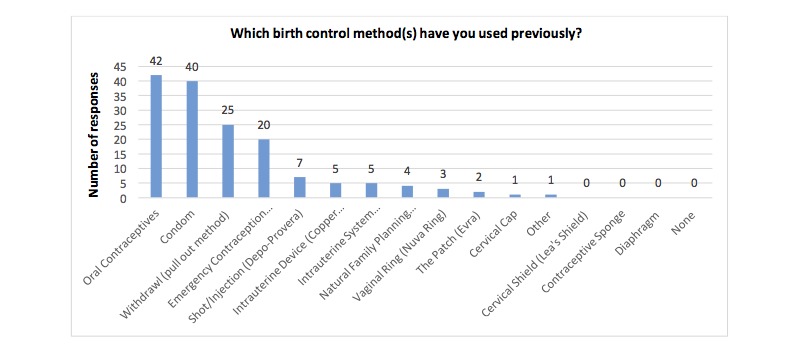
Previous contraceptive methods (numerical representation), n=50.

Figure 5 highlights previous contraceptive use. The top 2 methods were oral contraceptives, followed by condoms. Participants were asked if they were using a birth control method just before the index pregnancy and 48% (24/50) stated yes, 28% (14/50) said no, and 24% (12/50) said sometimes.

We further explored questions around contraceptive use and behavior using an adapted version of ORTHO Birth Control Satisfaction Assessment Tool [33]. We analyzed the questions according to lifestyle impact (63), compliance/adherence (51), and assurance/confidence on a scale of 0 to 100 (58). Higher scores indicate better satisfaction; however, the results ranged between 50 and 65, suggesting that participants were neutral with regard to the degree of satisfaction with previous contraceptive use. This information was used to assist with content and design elements for the mobile intervention.

### Semistructured Interviews

A thematic analysis approach was used to analyze the interviews. We explored themes around 3 specific categories: (1) participants’ overall abortion experience and how that may inform their thoughts on follow-up both for physical and/or emotional well-being, (2) current level of interaction with technology and specifically around health care–based issues, and (3) preferences for a mobile intervention to support follow-up care following a surgical abortion. Overall, 10 key themes were identified. These are listed in Textbox 1 with definitions of each on the basis of the various nodes and subnodes that were established from the analysis. Half of the participants who were interviewed traveled greater than 50 km to obtain care at 1 of the 3 urban abortion clinics. Importantly, they noted that for women such as themselves who travel far distances or are from rural areas, a resource that is comprehensive, secure, simple, and accessible is an important need and would address a gap in postabortion care.

**Definitions of themes on basis of nodes and subnodes. (Numbers represent the nodes and the letters [a, b, c...] represent the subnodes.)**

*Talking about abortion*

- Decision making
- Abortion experience at the clinic
- Stigma

*Accessing health information*

- Health professionals
- Internet
- Friends/family
- Other sources

*Barriers to health care*

- Time
- Appointments/wait times

*Contraception*

- Access
- Education
- Current and past use
- Motivation
- Side effects

*Desire for Postabortion counseling*

- Accessing counseling

*General Postprocedure needs*

- Physical
- Emotional
- Support networks

*Understanding follow-up*

- Definition
- Needed
- Desired

*Features and considerations of a mobile intervention*

- Design
- Content
  - Language
  - Topics
- Experience with previous mobile apps

*Features and considerations for a text messaging service*

- Content
- Convenience
- Frequency
- Preference

*Privacy and confidentiality*

#### Talking About Abortion

Participants unanimously described the experience as lonely, particularly as they felt that they were unable to talk to someone about the procedure. When asked if participants would want an intervention that allowed them to connect with other women or to resources where women share their stories, many supported this. One participant stated:

...I like the principle of women being able to talk about their experiences and I think that just creates more and more awareness, and awareness is a positive thing

Some felt that for religious or cultural reasons they may not feel comfortable speaking to their health care provider and that having a mobile intervention that was comprehensive and evidence based would be a good way to access information.

#### Accessing Health Information

The majority of our participants utilized Google as their first point to access information but did not view this as a panacea to health information. Ultimately, participants preferred to obtain information from a trusted health care provider. One participant stated:

...sometimes I would probably go online first and just Google things and try and find things out but I usually try and go to a doctor eventually. When I go online it’s just like premature before I decide to go to the doctor

Participants would also use the internet to elicit opinions of other women on birth control:

I Googled it [referring to the intrauterine device] to see what people thought of it. I do that with every kind of birth control. But at the same time you have to take it with a grain of salt because people don’t post reviews on contraception if they like it. They only post negative reviews.

This information was useful for our study team and key stakeholders when considering content development for contraception.

#### Barriers to Health Care

Participants provided valuable information to highlight that stigma around abortion continues to exist among health care providers and specifically in rural areas. This theme was valuable to consider when we engaged with our rural key stakeholders to share and elicit information from their perspective of the challenges of rural abortion care. One participant stated:

I live in a remote community. For sure, and it’s a really small town. So I probably wouldn’t even feel comfortable going to somebody because it’s a small town.

Another participant commented that there is not enough information on the abortion services provided in British Columbia on the internet, and rather, the first resources that are presented are pregnancy options services that are in fact centers to convince women to keep their pregnancies. This participant stated:

...because when I was looking it up on the Internet a bunch of places came up for pregnancy options but none for actual clinics that do these procedures and what not.

This highlighted that we needed to consider a resource that had a comprehensive and vetted list of resources that women could refer to.

#### Contraception

We asked participants about their contraceptive behaviors and preferences because this is a component of abortion care and subsequent follow-up. Participants shared their frustration that contraception is not free in Canada. Furthermore, concerns about good access were brought up frequently, as was the need to be well informed that contraception is not just for pregnancy prevention but also for prevention of sexually transmitted infections. Participants were motivated to use contraception to prevent future pregnancies.

#### Desire for Postabortion Counseling

This was a consistent theme among participants. Many stated that it would be useful to have access in person as well as remotely to postabortion counseling. One participant who had her second procedure had used counseling postabortion after her first termination and stated:

when I first got a termination years ago, I was super against it [counselling] because I was an idiot and I was 20. And then I did it anyway and it was the best thing I could have ever done.

#### General Postprocedure Needs

We asked participants about their general postprocedure needs. The vast majority felt good physically but found more emotional hardships. One participant stated:

Physically I felt better than I expected. I was in bed for maybe a day, but it wasn’t bad. It wasn’t painful. Like, I have bad cramps on my period and it was at most like that. But that only lasted a short time. But otherwise the pregnancy symptoms left pretty quickly, I guess in about a week, some of them sooner than that.

On the emotional experience, a participant stated:

Emotionally it was harder than I expected it to be. I knew that this was something I needed to do if this was to happen. But I never obviously had to do it before, so it was definitely harder, even though I knew it was the right thing to do. It still brought up a lot of emotional questions and feelings.

Another participant highlighted the importance for a postprocedure intervention to include a phone call or text message that would be a check-in with the patient. Many stated it was an isolating and lonely experience and to have either a service that connected them to other resources and services or a live chat group would be something valuable.

#### Understanding Follow-Up

Despite follow-up being an important aspect of health care, there are little data published that examine how individuals perceive follow-up. This is important to consider when developing a postabortion support tool. When asked what follow-up means, a participant stated:

...with a healthcare provider, I guess just following up with any physical, medical relevant issues.

Furthermore, some participants expressed desiring follow-up based on whether or not they had an intrauterine device inserted at the time of abortion. One participant stated:

They just told me to come back in six weeks to get an ultrasound of my IUD to make sure it’s in the right place...and I’m assuming they’ll probably see how I’m feeling emotionally, physically and—that was my understanding of the follow-up.

When we asked participants whether they felt follow-up was needed and/or desired, a vast majority stated that it would be desired for emotional support, and they did state it was a necessity physically as that was what was told to them at the time of their procedure. However, many had recovered physically a few days after and noted that emotional and social support was desired. Some also commented that it might be good to have a mobile intervention that allowed women who lived in remote communities to have access to. The following are excerpts from individuals who highlighted the desire for emotional support and the benefit of a secure resource for those living in rural areas.

I feel like it’s a—like, for mental health I feel like maybe it’s more desired

I think it would probably be beneficial for more people who are living in more rural or conservative communities who might not feel comfortable talking to their doctor or don’t have very good access to the aftercare. I’m fine ’cause I’m in downtown Vancouver. But other people might not have such an easy time.

#### Features and Considerations of a Mobile Intervention

Participants consistently stated that they wanted an easy-to-navigate, simple, professional mobile intervention that was concise and user-friendly. In addition, 2 of our participants expressed that it should be:

Easy to navigate...more simple, straightforward but with options to look at more information if you wanted some

and:

I think just simplicity and user-friendly ease sort of is important. Not having too much information which to be honest I don’t know how you do it because there’s a lot of information on this topic.

Participants also stated that the intervention should allow them to unsubscribe, particularly, if it included text or email notifications. There was also encouragement to include partners in the intervention and to have information relevant for partners specifically. One participant commented on the importance to ensure that the app or website is functioning and checked frequently. She stated:

Mediocre apps are just an inconvenience, and I feel like this category, it’s not—I don’t know, I feel like it just wouldn’t get the funding that it needed for it to really work properly. I have no patience for apps that don’t work well. So if it’s not awesome, it’s not on my phone and I’m not wasting my time using it.

Participants suggested that options listed in the form of a dropdown list would allow for ease of navigation. Participants also wanted to be able to book counseling services directly online with the counselors from the respective abortion clinics where they received services. This was a service already provided by the counselors but participants encouraged this be part of a follow-up mobile technology resource. Finally, having more concise, patient-centered information on contraception in an easy-to-access manner was an expressed need:

More information on contraception and all the different types that women can use because I know a lot of my girlfriends the only type they use is a condom or birth control...I feel like there should be more information on the IUDs because it would be a really good alternative for some women who are like me and would prefer just to not have to worry about taking oral contraceptives at a certain time if they’re busy or something like that. Because my current experience with birth control is after going to the clinic and receiving the information that I got, I realized the IUD was best for me. And there should be more information about that, whether it be on an app or on the Internet.

#### Features and Considerations for a Text Messaging Service

Overall, participants encouraged follow-up by either an individual from the clinic texting them at set times or an email or text messaging service that was automatic and timed in appropriate frequencies. It was important that there be an unsubscribe feature. Furthermore, participants stated that weekly messages at first would be useful but then to decrease these messages to monthly up to a maximum of 6 months. The following excerpts highlight this theme:

I think it’s a really good thing. I would definitely have somebody remind me about appointments, remind me to get a checkup,...I would definitely use that. But I think as well—as long as you consent to it and you can set the frequency yourself, like, I think it’s a great idea.

I think that would be handy as long as it had the option to stop at any time, an unsubscribe feature

#### Privacy and Confidentiality

Privacy and confidentiality was very important for women who were interviewed, which was slightly different from the results of the quantitative survey. Participants stated that they would want something that is discrete and did not mention the words abortion or have any way to link the patient to the clinic where they had their procedure:

I think confidentiality is a big part of it, just because—especially with some people, they don’t really want everyone to know. I know I don’t want everybody in the whole world to know what happened.

Privacy is everything for me. That’s the most important thing for me. And I think that’s maybe because I do live in a small town. It’s really, really important to have that here. If you’re in a big anonymous city it might be different.

Obviously with mobile technology you want to be really considerate of privacy and have secure servers and—especially if there’s contact information with a whole list of women...as it relates to mobile, that’s definitely something to consider.

## Discussion

This study represents the first phase of a 3-phase study and highlights the importance of formative research that incorporates the voice of end users to assist with development of a mobile intervention for follow-up after an induced surgical abortion. Specifically, this formative mixed-methods study explored women’s interactions with technology and important aspects to design and content for a mobile intervention to support follow-up after a surgical abortion. It is important to include a formative aspect to the development of technology-based interventions to improve women’s health generally, but abortion care specifically [20,36,38-40]. The theoretical framework for all 3 phases included both the TAM and TRA theories. In this study particularly, we were able to elicit information about what would be important in the design of the tool, what are the contextual factors to consider in designing the tool but also around the stigma women continue to experience around abortion and how that can inform one’s attitude and intentions to engage in a tool to support them in care related to an abortion. This information was utilized in the development of the intervention and further testing for phases II and III.

According to the literature, including the voice of end users early in the development of an intervention is a fundamental principle of human-centered design [16,20,38,39]. A study conducted by Smith et al [20] explored women’s needs in Cambodia to develop a mobile phone–based intervention to support postabortion family planning, specifically, contraceptive adherence [20]. Similarly, researchers from University of San Francisco’s Program in Women-Centered Contraception, developed a tablet-based contraceptive decision support tool for women [41]. This study utilized a multiphase approach that incorporated the end user throughout the entire design of the project. The formative research findings from these studies emphasized that using an iterative process informed by patient and provider input throughout the development and testing led to a more patient-centered innovation [42]. In addition, the formative research in these studies identified main patterns of mobile use of the women from the respective countries, main reasons and expectations for contraception and abortion care, researchers drew on components of existing interventions and behavior change theories to then develop conceptual frameworks. It also highlights that anytime a mobile intervention is to be adapted to a new context, formative research is essential to ensure the tool can be adapted to particular contexts and cultures.

In this study, a theme that resonated was the desire for a tool that incorporated emotional support as part of follow-up abortion care, which has been previously described in the published literature. A qualitative study conducted in Canada explored women’s abortion experiences in the Yukon territory, a remote service area, highlighting that “fragmented services left women unsatisfied, stressed and upset about lack of information, multiple appointments and lengthy wait times” [43]. Women further expressed frustration with lack of follow-up counseling and recommended that it be routinely offered as they feel contact with health care providers is cut off after the procedure [43]. In addition to access issues, barriers of cost, knowledge among the general public, health care provider competence, and attitudes have also been highlighted in the literature [44]. Another study explored women’s expressed desire for postabortion support services, highlighting the stigma around abortion that exists in political and social contexts, preventing women from sharing their experiences [1]. This study specifically highlighted that though women may not necessarily need mandatory physical follow-up, they desire access to postabortion support for emotional well-being [1]. Furthermore, there is great deal of inconsistency in the type of support and information available to women after an abortion. In addition to emotional support, participants stressed key characteristics of the mobile intervention such as privacy and confidentiality, evidence-based information, some form of interaction with the health care system, and is professional and easy to use.

Finally, it was important to capture the voice of women who traveled from rural and remote areas. According to our demographic data, over one-third of our participants traveled over 50 km to obtain an abortion. Though abortion is legal in Canada, there are access barriers, particularly for women living in remote and rural areas. Studies that have developed strategies in restrictive settings utilizing mHealth could be adapted to rural and remote contexts that have restricted access to abortion services in Canada. Providing safe and effective means, such as telemedicine, text messaging services, or mobile apps, has been proven to be acceptable and satisfactory for women in legally restricted settings [12,21-23,26] and, therefore, could have potential for Canadian women living in rural and remote areas, where abortion is legal but barriers to service delivery exist.

The limitations for this study include overall generalizability to other populations and small convenience sample sizes. Furthermore, abortion continues to be stigmatized, which can contribute to difficulties with recruitment and loss to follow-up. Accordingly, we noted that recruitment for the qualitative interviews took longer than expected and further assumed that lack of participant engagement may be associated with conducting research a few weeks out after the abortion, where participants have moved on and are resuming back in their busy lives. Consideration of recruitment strategies will need to be taken into consideration for future studies, particularly when thinking about diversifying the participants recruited and obtaining robust response rates for analysis.

Balancing these limitations are the strengths of our study, including user engagement early in the research design, a mixed-methods design incorporating both quantitative and qualitative data, engagement with both urban and rural key stakeholders, and robust findings that will inform the design of the mobile intervention.

Details of phases II and III will be published as separate papers. Specifically, phase II incorporated these findings to develop, design, and test an intervention based on findings from this study, and phase III was a pilot, prospective mixed-methods study to determine the feasibility and acceptability of the tool for women who undergo a surgical abortion.

### Implications

This study was the first phase of 3 and perhaps the most important phase as it determined crucial findings about women’s interactions with technology and their preferences for design and content of an intervention that could support their care after a surgical abortion. Moreover, there is great deal of momentum toward self-managed abortion care and technology has a role to play. This study is the first to utilize mHealth and user-centered design in Canada as a novel approach to provide a means for women to self-manage their follow-up care after a surgical abortion.

## Abbreviations

mHealth: mobile health
TAM: technology acceptance model
TRA: theory of reasoned action
